# Analysis of Mechanical Properties of Fiber-Reinforced Soil Cement Based on Kaolin

**DOI:** 10.3390/ma17092153

**Published:** 2024-05-04

**Authors:** Junnan Zhao, Zhongling Zong, Hang Cen, Pai Jiang

**Affiliations:** School of Civil and Ocean Engineering, Jiangsu Ocean University, Lianyungang 222005, China; z18326688872@163.com (J.Z.); , rex.pai.jiang@gmail.com (P.J.)

**Keywords:** fiber-reinforced soil cement, unconfined compressive strength, splitting tensile strength, polypropylene fibers (PPFs), polyvinyl alcohol fibers (PVAFs), glass fibers (GFs)

## Abstract

Adding fibers into cement to form fiber-reinforced soil cement material can effectively enhance its physical and mechanical properties. In order to investigate the effect of fiber type and dosage on the strength of fiber-reinforced soil cement, polypropylene fibers (PPFs), polyvinyl alcohol fibers (PVAFs), and glass fibers (GFs) were blended according to the mass fraction of the mixture of cement and dry soil (0.5%, 1%, 1.5%, and 2%). Unconfined compressive strength tests, split tensile strength tests, scanning electron microscopy (SEM) tests, and mercury intrusion porosimetry (MIP) pore structure analysis tests were conducted. The results indicated that the unconfined compressive strength of the three types of fiber-reinforced soil cement peaked at a fiber dosage of 0.5%, registering 26.72 MPa, 27.49 MPa, and 27.67 MPa, respectively. The split tensile strength of all three fiber-reinforced soil cement variants reached their maximum at a 1.5% fiber dosage, recording 2.29 MPa, 2.34 MPa, and 2.27 MPa, respectively. The predominant pore sizes in all three fiber-reinforced soil cement specimens ranged from 10 nm to 100 nm. Furthermore, analysis from the perspective of energy evolution revealed that a moderate fiber dosage can minimize energy loss. This paper demonstrates that the unconfined compressive strength test, split tensile strength test, scanning electron microscopy (SEM), and mercury intrusion porosimetry (MIP) pore structure analysis offer theoretical underpinnings for the utilization of fiber-reinforced soil cement in helical pile core stiffening and broader engineering applications.

## 1. Introduction

Soil cement is a material produced by mixing, stirring, and hardening cement, soil, and other components in appropriate proportions. Cement blending ratios are generally determined by the soil cement performance specifications required by the project and typically range from 10 to 20% cement [1]. As a widely used material in the field of construction and civil engineering, soil cement plays a vital role in slope reinforcement, pit support, and foundation treatment [2,3,4]. However, in practical engineering applications, soil cement predominantly exhibits brittle damage. The addition of fibers can effectively improve the ductility and reduce the brittleness of soil cement, transforming its damage profile from brittle to ductile or plastic, and enhancing its deformation capacity.

In recent years, researchers worldwide have incorporated various fibers into soil cement to enhance its mechanical properties. Maher et al. [5] investigated the effects of doping polypropylene fibers and glass fibers into kaolinite clay. They found that the unconfined compressive strength of kaolinite clay increased with the increase in fiber dosage. The unconfined compressive strength of kaolinite clay reached its maximum when the fiber dosage was 5%, showing an increase of 1.3 times and 1.55 times, respectively. Gupta et al. [6] determined through the California Bearing Ratio (CBR) test that the addition of a 10% mass fraction of rice husk ash resulted in a CBR value six times higher than the initial CBR value. Sukontasukkul et al. [7] investigated the effects of incorporating polypropylene fibers and steel fibers into fiber-reinforced soil cement at various volume fractions. They verified that the equivalent flexural strength ratio and residual strength of the fiber-reinforced soil cement increased with fiber incorporation. Kumar et al. [8] conducted unconfined compressive strength tests on plain and bent polyester fiber–clay specimens doped with different dosages and lengths. The results demonstrated that the incorporation of fibers into the mix led to an increase in the equivalent flexural strength ratio and residual strength, thus enhancing the flexural properties. Notably, polypropylene fibers exhibited superior performance compared to steel fibers. Moreover, toughness increased with the rise in volume fraction. Park [9] investigated the variation in fiber-reinforced soil cement strength with fiber distribution by conducting a series of unconfined compressive strength tests. The results indicated that the strength of the specimen with fibers uniformly distributed in five layers was twice that of the non-fiber-reinforced specimen. In addition, some studies have shown that the strength of soil cement specimens reaches a certain peak at a certain fiber dosage [10,11,12,13]. Prabakar and Sridhar [14] selected sisal fibers as reinforcement and conducted compaction and triaxial compression tests. They observed that the shear stress increased nonlinearly with fiber length and decreased with increasing fiber content, particularly when the fiber content exceeded 0.75%. Buathon et al. [15], through their study on the unconfined compressive properties of palm fiber-reinforced cement-stabilized sand, concluded that the optimal performance of cement sand occurred at a fiber dosage of 1.0% and a fiber length of 40 mm. Xu et al. [16] investigated the impact of rice husk fiber dosage, cement dosage, and maintenance period on the strength of soil cement doped with rice husk fiber. They conducted both unconfined compressive strength tests and straight shear tests. The results indicated that the soil strength peaked with 12% cement and 2% rice husk fiber dosage after 60 days of maintenance. Additionally, the cohesive strength of the soil was highest with the same cement and rice husk fiber dosages.

Clay within the cementitious matrix not only significantly enhances the mechanical strength and durability of the material but also optimizes its microstructure and enhances its environmental adaptability. These enhancements not only render the cementitious materials more stable and reliable in harsh environments but also directly reduce the carbon footprint through decreased cement usage, thereby promoting the environmental and sustainable development of construction materials [17].

**Figure 1:**
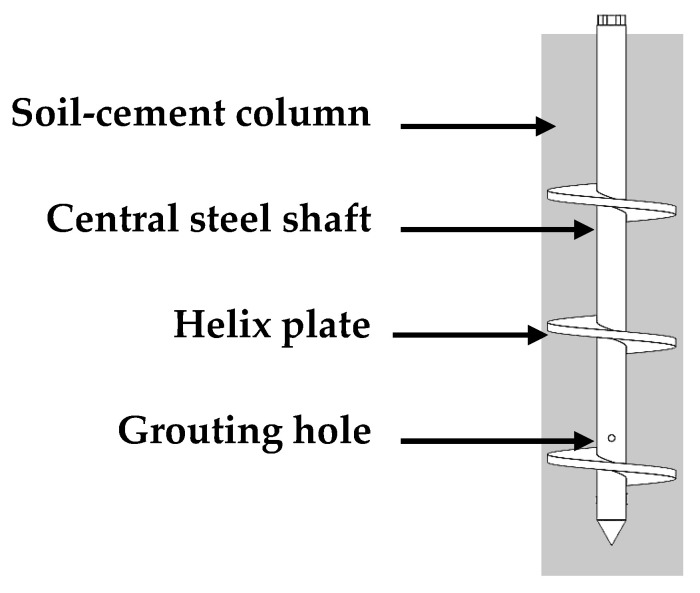
The Helix-Stiffened Cement Mixing piles.

Strength composite piles are created by inserting rigid core piles into soil cement piles or by drilling holes in soil cement piles and subsequently pouring concrete or reinforced concrete. This process results in a unified pile structure comprising rigid core piles encased in soil cement, also referred to as “strength composite piles” [18,19]. Pressure grouting helical piles employ grouting technology, with synchronous grouting occurring during the drilling of steel pipe helical piles. The slurry is injected into the helical pile’s hollow part through the grouting pipe, and then expelled through grouting holes. The helical blade mixes the slurry with the soil, forming soil cement around the pile periphery [20,21]. The Helix-Stiffened Cement Mixing piles are shown in Figure 1. The incorporation of steel fibers into concrete piles can effectively enhance their ductility and isotropic bearing capacity [22,23]. Existing research has extensively explored the application of fibers in cementitious materials, yielding significant findings on the mechanical properties of fiber-reinforced soil cement. Currently, the global research and application of fiber-reinforced soil cement predominantly focus on formulations with low cement content. Studies on fiber-reinforced soil cement with higher cement incorporation ratios are less frequent. Fiber blended into cement, when applied in Helix-Stiffened Cement Mixing piles, forms fiber-reinforced soil cement Helix-Stiffened Cement Mixing piles that can mitigate the pile foundation’s susceptibility to brittle damage in actual projects. However, during pile formation, the precise soil cement ratio remains uncertain due to the non-uniform soil formation around the pile, influenced by grouting pressure and the speed of drilling. This paper presents innovative research by studying fiber-reinforced soil cement with higher cement mixing ratios, which more closely approximates actual engineering conditions. It provides theoretical references for the development and application of Helix-Stiffened Cement Mixing piles in subsequent projects.

## 2. Materials and Methods

### 2.1. Materials

**Figure 2:**
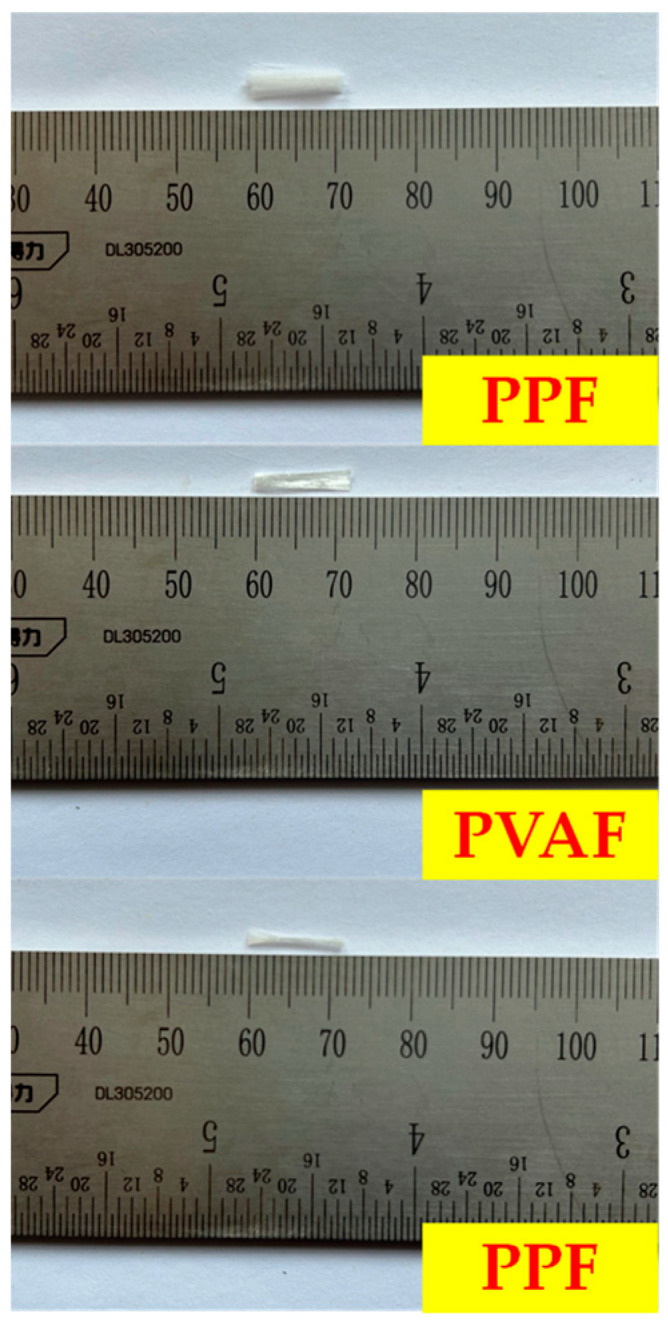
Three fiber lengths.

The soil sample used in this test was kaolin, originating from Jingdezhen, Jiangxi Province, China, and conforming to the Chinese standard for geotechnical testing methods (GB/T50123-2019) [24]. Its physico-mechanical indices are detailed in Table 1. In this experiment, three types of fibers were selected: polypropylene fiber (PPF), polyvinyl alcohol fiber (PVAF), and glass fiber (GF), originating from Weifang, Shandong Province, China. The physical and mechanical properties of these fibers, each with a length of 12 mm, are detailed in Table 2. Three fiber lengths are shown in Figure 2. The cement used in this test was PO42.5 ordinary silicate cement, originating from Shanghai, China, and conforms to the Chinese standard common Portland cement (GB175-2020) [25]. Its physico-mechanical parameters are detailed in Table 3.

### 2.2. Specimen Preparation

Prior to the experiment, the soil designated for testing was placed in an oven at 110 °C for 24 h. Following drying, the soil was removed, passed through a 2 mm sieve, and then transferred into a bucket and sealed with plastic wrap for preservation, which helps avoid the absorption of moisture from the air. A fixed water–cement ratio of 0.6 was maintained, with the cement constituting 75% and the dry soil 25% of the mixture’s mass. PPFs, PVAFs, and GFs were blended with the mixture of cement and dry soil in mass fractions of 0%, 0.5%, 1.0%, 1.5%, and 2.0%. Refer to Table 4 for detailed information.

**Figure 3:**
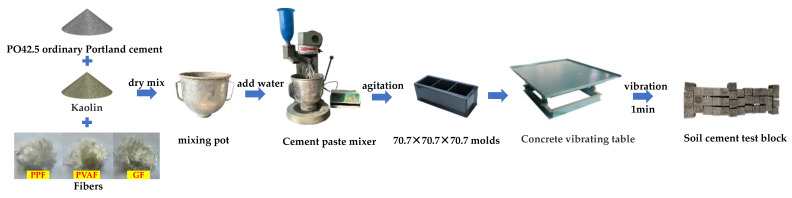
Specimen making process.

Initially, the reserved test soil was combined with cement for approximately 1 min; after blending the cement and dry soil, fibers were incorporated according to the designated proportion and blending continued for approximately 1 min. Subsequently, water was added and mixing continued for about 2 min. Given the challenge of dispersing fibers in the mixture, they were manually separated prior to the experiment. The observation of fiber dispersion occurs during the dry mix stage; should fiber clumping be detected, manual separation is applied as needed. The blended mortar was transferred into a 70.7 × 70.7 × 70.7 mm^3^; cube mold, which had been pre-coated with machine oil to facilitate demolding post casting [26]. Once filled, the mold was positioned on a vibration table and vibrated evenly for 2 min to remove air bubbles from the specimen; the surface was then smoothed with a scraper. Following vibration, the mold was laid on a level indoor surface and covered with plastic wrap to inhibit water evaporation. Specimens were demolded and labeled following 24 h of maintenance at room temperature, and then placed in a standard curing chamber for 28 days post demolding. To guarantee test accuracy, each specimen group consisted of 6 samples, totaling 78, employed for soil cement unconfined compressive strength tests and soil split tensile strength tests, with results averaged [26]. The process of specimen production is illustrated in Figure 3.

### 2.3. Unconfined Compressive Strength Test

The dimensions of the specimen for the unconfined compressive test were 70.7 mm × 70.7 mm × 70.7 mm (cubic). This test employs a WDW-100L type electronic universal testing machine, from Changchun, Jilin Province, China, utilizing displacement loading at a rate of 1 mm/min and continuing until the specimen fails. The peak load value was recorded to determine the unconfined compressive strength [27]. Three specimens underwent testing for unconfined compressive strength, and the results were averaged.

### 2.4. Splitting Tensile Strength Test

The determination of tensile strength typically involves either a direct tensile test or a split tensile strength test. The former is more complex, and the size of the specimen significantly impacts the test results. Compared to the direct tensile test, the split tensile strength test offers a simpler procedure. Therefore, this study utilized the split tensile strength test to assess the tensile strength of fiber soil cement [28]. The test employs a WDW-100L type electronic universal testing machine, with a specimen size of 70.7 mm × 70.7 mm × 70.7 mm (cubic), and utilizes the same loading method as the unconfined compressive strength test. Prior to the test, the specimen was positioned in the mortar-splitting tensile fixture, and then placed between the press’s upper and lower pressure plates. The load was applied once tight contact was achieved, and the destructive load was recorded for conversion into the splitting tensile strength [26]. Three specimens were subjected to the split tensile strength test, with the results subsequently averaged. The concrete splitting tensile strength was calculated using the following equation [29]:

(1) $$f_{ts}=\frac{2P_{\max}}{\pi a^{2}},$$

where $f_{ts}$ is the splitting tensile strength of soil cement; $P_{\max}$ is the maximum load value when the specimen is damaged; and a is the side length of the specimen.

### 2.5. Scanning Electron Microscope Test and MIP Test

The SEM (scanning electron microscope) test was conducted using a JEOL (Japan Electronics, Toshiba, Japan) JSM-7800F thermal field emission scanning electron microscope. After ion sputtering for 1 min, a vacuum test was performed on the apparatus.

Mercury intrusion porosimetry, widely utilized for examining the pore structure of materials, received approval for soil and rock analysis in 1984. Although initially not applied to cementitious materials, it is now employed across a broad spectrum of materials and fields [30]. In 1921, Washburn introduced a method for analyzing pore structure, involving the compression of a non-wetting liquid into a porous solid. Mercury, a non-wetting liquid at room temperature, penetrates pores only under specific pressures. The volume of mercury injected is recorded alongside pressure variations, allowing pore size to be inferred from the applied pressure using the Washburn equation [31].

(2) $$D=-\frac{4\gamma \cos\theta }{P},$$

where D is the radius of the pore; P is the pressure required to enter the pore to overcome the resistance; θ is the wetting angle between the pore and the material; and γ is the interfacial tension between mercury and air.

In this study, mercury intrusion porosimetry was conducted using a fully automated Mercury Piezometer, model AutoPore V9600, from Micromeritics Instrument Corporation, Norcross, GA, USA. The expansion meter featured a bubble with a volume of 5 cm³ and a total capacity of 1.716 cm³, with the sample prepared to occupy the majority of this volume. Testing commenced with low-pressure measurements ranging from 0.014 MPa to 0.207 MPa, followed by high-pressure analysis from 0.276 MPa to 206.843 MPa.

This paper conducted SEM and mercury intrusion porosimetry tests on fiber-reinforced soil cement specimens with varying fiber dosages. Samples for SEM and mercury intrusion porosimetry tests were collected after curing the fiber-reinforced soil cement specimens for 28 days. Before MIP testing, specimens must be dried thoroughly to ensure that the pores are open for intrusion.

## 3. Results and Discussion

### 3.1. Unconfined Compressive Strength

**Figure 4:**
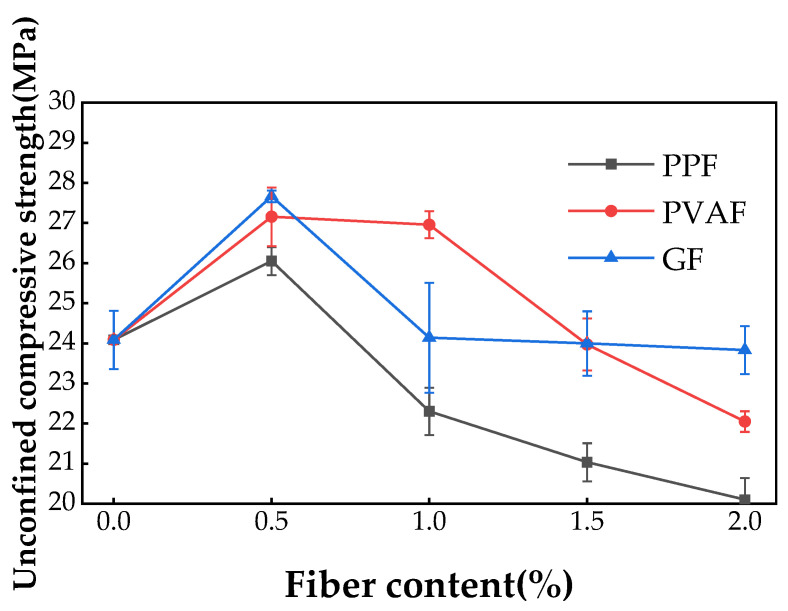
Unconfined compressive strength of three types of fiber-reinforced soil cement with different fiber dosages.

Figure 4 displays the results of the unconfined compressive strength test. The unconfined compressive strength of PPF-, PVAF-, and GF-reinforced soil cement exhibits an initial increase followed by a decrease, peaking at a fiber content of 0.5%, with respective strengths of 26.72 MPa, 27.49 MPa, and 27.67 MPa. The unconfined compressive strength increased by 10.9%, 14.1%, and 14.9%, respectively, over plain soil cement specimens. The GF-doped specimens had the highest unconfined compressive strength. The increase in unconfined compressive strength for the GF-doped specimens was also the greatest. Upon exceeding a 0.5% fiber content, the unconfined compressive strength of all three types of fiber-reinforced soil cement declined. For the PPF-doped specimens, a 1% fiber content resulted in unconfined compressive strength lower than that of the plain soil cement; at a 2% fiber content, this strength further decreased to 20.10 MPa. The PVAF-doped specimens experienced a minor decline in unconfined compressive strength from a 0.5% to a 1% fiber content, and at a 2% fiber content, it decreased to 22.39 MPa, lower than the strength of the plain soil cement. The GF-doped specimens showed a significant decrease in unconfined compressive strength from a 0.5% to a 1% fiber content, with a less pronounced decrease beyond 1%, ultimately reducing to 23.50 MPa, slightly below the strength of plain soil cement. Khattak [32] demonstrated that the combined reinforcement of soft soils with polypropylene fibers and cement can mutually enhance their properties. Jiang et al. [33] established that within the effective range of fiber admixture ratios, the internal friction angle and cohesion of the soil body will increase significantly with the increase in the fiber admixture ratio; these findings are consistent with the results of this paper.

**Figure 5:**
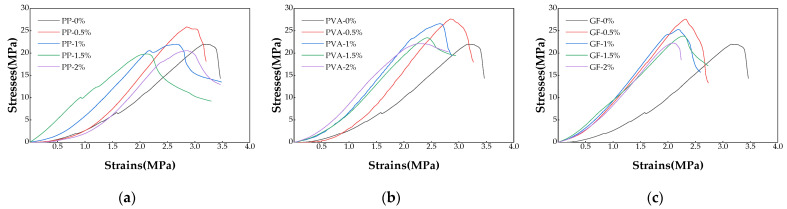
Stress–strain curves of three types of fiber-reinforced soil cement with different fiber dosages. (**a**) PPF-reinforced soil cement stress–strain curve, (**b**) PVAF-reinforced soil cement stress–strain curve, and (**c**) GF-reinforced soil cement stress–strain curve.

Figure 5 displays the stress–strain curves for the three types of fibers at varying dosages, as determined by the unconfined compressive strength test. According to the first law of thermodynamics, the increase in an object’s internal energy is equal to the sum of the heat it absorbs and the work performed on it. During the unconfined compressive strength test, the external load applied by the testing machine deforms and damages the soil cement. The process of destruction entails a mutual conversion of energy. Assuming no energy exchange between the soil cement and its surroundings during the damage process, constituting a closed system, the work performed by the testing machine’s axial compression on the soil specimen equates to the stored elastic strain energy during its elastic deformation and the energy dissipated through plastic deformation and internal cracking [34], as represented by the following equation:

(3) $$W=W_{e}+W_{d},$$

where W is the work performed on the soil specimen by the axial compression of the testing machine; $W_{e}$ is the elastic strain energy that can be stored during the elastic deformation stage of the soil cement specimen; and $W_{d}$ is the dissipated energy generated by plastic deformation and cracking within the soil cement specimen.

The work performed on the specimen by the testing machine’s axial compression, along with the elastic strain energy stored during the soil cement specimen’s elastic deformation phase, can be articulated as follows [35]:

(4) $$W=\int \sigma_{1}d\varepsilon_{1}=\sum_{i=0}^{n-1}\frac{1}{2}\left(\varepsilon_{i+1}-\varepsilon_{i}\right)\left(\sigma_{i+1}-\sigma_{i}\right),$$

(5) $$W_{e}=\frac{1}{2}\sigma_{1}\varepsilon_{1}\approx \frac{\sigma_{1}^{2}}{2E},$$

where $\sigma_{1}$ is the principal stress of the soil cement specimen; $\varepsilon_{1}$ is the strain value of the soil cement specimen; $\sigma_{i}$ is the stress value at any point of the stress–strain curve; $\varepsilon_{i}$ is the strain value at any point of the stress–strain curve; and E is the modulus of elasticity of the soil cement specimen, i.e., the slope of the straight line segment of the stress–strain curve.

**Figure 6:**
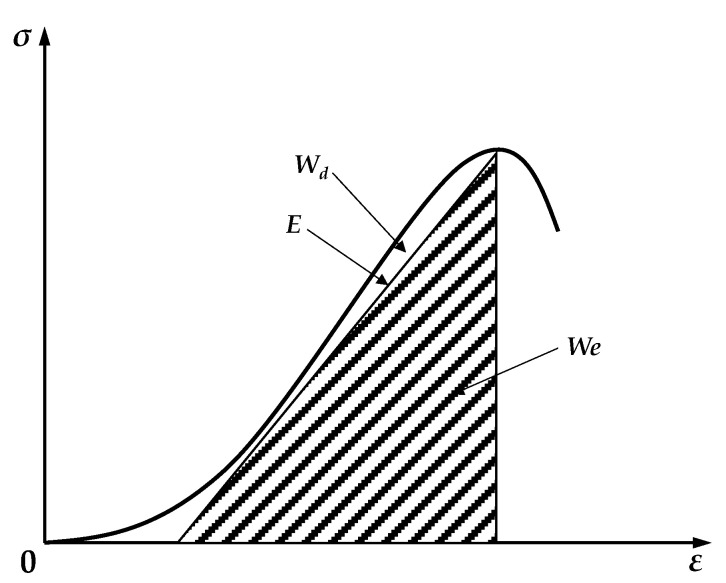
Figure of energy exchange during soil cement destruction process.

Figure 6 presents a schematic representation of the energy exchange during the destruction of soil cement as outlined above. The work performed on the soil cement specimen by the testing machine’s axial compression is depicted as the area of the curved-edge trapezoid in the figure, specifically, the area under the curve at the stress–strain curve’s peak. The elastic strain energy is represented by the shaded area in the figure, specifically, the triangular area along the straight-line segment of the stress–strain curve. The difference between these two areas represents the dissipated energy.

**Figure 7:**
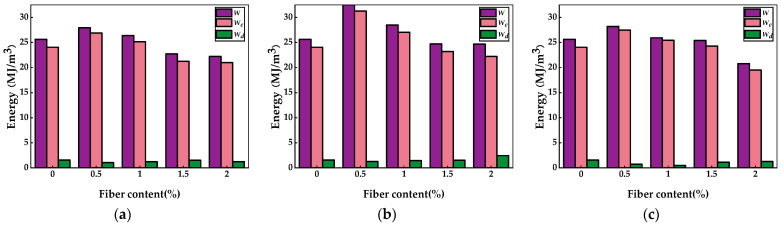
Energy parameters of fiber-reinforced soil cement. (**a**) PPF-reinforced soil cement energy parameters, (**b**) PVAF-reinforced soil cement energy parameters, and (**c**) GF-reinforced soil cement energy parameters.

The energy parameters for fiber-reinforced soil cement are derived from stress–strain curve calculations, as detailed in Table 5 and Figure 7. The data in Table 5 reveal that the total energy and elastic strain energy of the three types of fiber-reinforced soil cement initially increased and then decreased with the fiber dosage, peaking at a dosage of 0.5%. Respectively, the total energy values were 27.93692 MJ/m³, 32.53887 MJ/m³, and 28.20924 MJ/m³; the elastic strain energy values were 26.88218 MJ/m³, 31.26096 MJ/m³, and 27.47410 MJ/m³. This trend aligns with the results from the unconfined compressive strength tests, where PVAF-reinforced soil cement exhibits the highest total and elastic strain energy, with dissipated energy minimizing at a fiber dosage of approximately 0.5%. Liu et al. [36] observed that the total energy and elastic strain energy of soil cement initially increased and subsequently decreased with the addition of polypropylene fiber content during the unconfined compressive strength test. This observation aligns with the results presented in the current study. The analysis indicates that appropriate fiber incorporation can enhance the total and absorbed elastic strain energy of soil cement, reduce dissipated energy, and minimize energy loss. This improvement is attributed to the fibers’ bridging effect in soil cement, which inhibits crack formation and development and mitigates stress concentration [37].

### 3.2. Splitting Tensile Strength

**Figure 8:**
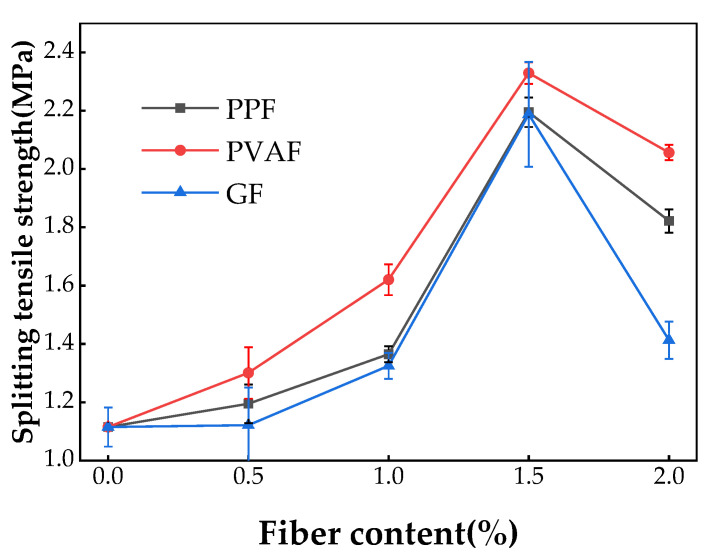
Split tensile strength of three types of fiber-reinforced soil cement with different fiber dosages.

Figure 8 displays the results of the splitting tensile strength test. The splitting tensile strength of PPF-, PVAF-, and GF-reinforced soil cement exhibited an initial increase followed by a decrease, peaking at a fiber content of 1.5%, with respective strengths of 2.29 MPa, 2.34 MPa, and 2.27 MPa. The split tensile strength increased by 106.3%, 110.8%, and 104.5%, respectively, over plain soil cement specimens. The split tensile strength of the PVAF-doped specimens was the highest. The growth rate in split tensile strength for the PVAF-doped specimens was also the greatest. Upon exceeding a 1.5% fiber content, the splitting tensile strength of all three types of fiber-reinforced soil cement declined, reducing to 1.91 MPa, 2.07 MPa, and 1.31 MPa, respectively, at a 2% fiber content. Despite this reduction, the splitting tensile strength of all fiber-reinforced variants remained higher than that of plain soil cement across all fiber dosages. The PVAF-doped specimens exhibited higher splitting tensile strengths than those reinforced with the other two fibers at all dosages. The GF-doped specimens experienced a negligible increase in split tensile strength at a 0.5% fiber content and a rapid decrease after exceeding a 1.5% fiber content. Zhao et al. [38] analyzed the compressive and splitting tensile strengths of net cement mortar and soil cement cubes with basalt fibers, discovering that these strengths first rose and then fell as fiber volume increased from 0% to 2%. This finding is consistent with the results of this paper.

### 3.3. Scanning Electron Microscope Analysis

**Figure 9:**
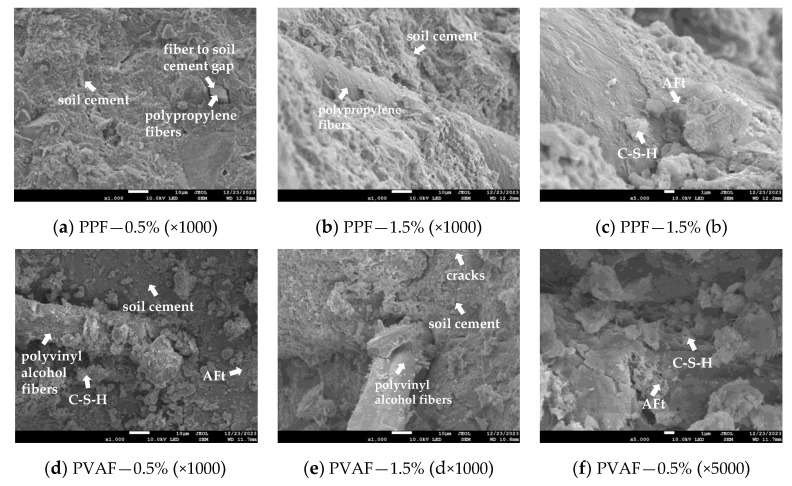

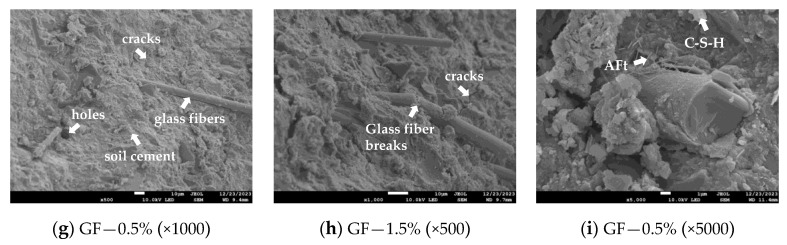
SEM images of the three fiber distributions.

Fiber-reinforced soil cement achieves reinforcement through bond strength, adhesion, and static friction between the fiber surface and the soil matrix [39]. Figure 9 displays the SEM images depicting the distribution of fibers in three types of fiber-reinforced soil cement: PPF, PVAF, and GF.

**Figure 10:**
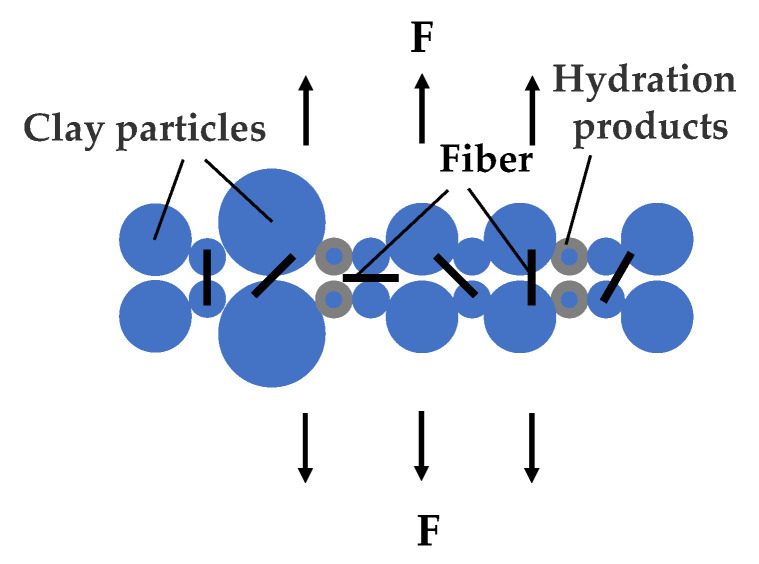
Figure of random distribution of fibers.

Figure 9a reveals a noticeable gap between PPF and soil cement, diminishing the bond strength between the fiber and the soil cement matrix, thereby undermining the reinforcing function of the fiber. Figure 9b demonstrates that PPF is tightly encased by soil cement, allowing the bond strength, adhesion, and static friction between the fiber and soil cement to fully manifest, indicating that these interactions significantly enhance the reinforcement provided by the fibers. Figure 9d,e show PVAF fibers bonding together, resulting in an uneven fiber dispersion, known as agglomeration. This indicates that an excess of fibers or their random distribution can lead to contact or even clumping among fibers. The smooth surface of these fibers, lacking adhesion, further reduces the contact area with the soil cement matrix and diminishes bond strength [40,41]. In Figure 9h, the glass fibers are shown to have fractured, suggesting that fractures occurred during the fabrication of the soil cement specimen. Additionally, during testing, external forces cause further fiber fracturing, diminishing the fibers’ bridging effect within the soil cement. Adding fibers can fill voids in the soil cement, enhancing the connection within the soil matrix, thereby strengthening its spatial structure and reinforcing its overall strength. However, fiber addition also leads to the creation of voids in the soil cement, as evident in Figure 9g, largely due to the fibers being pulled out. Given the fibers’ random distribution, their orientation within the soil cement is also random, rendering their reinforcing effect incomplete during testing, and may even introduce negative effects. Figure 10 illustrates fibers randomly distributed at various angles within the soil cement matrix, enveloped by clay particles and cement hydration products. Under external tension, fibers contribute differently based on their orientation, with some angles proving ineffective. Yet both the experimental setup and real-world applications are more complex than depicted, limiting the full potential of fiber reinforcement.

The hydration process of silicate cement constitutes a non-homogeneous and highly complex multiphase chemical reaction. Upon water addition, the ongoing hydration reaction transforms dispersed cement powder particles into a bonding cement paste, generating various hydration products that effectively unify particles of differing sizes. The primary hydration reaction products include calcium aluminate (AFt) with high crystallinity and amorphous hydrated calcium silicate (C-S-H) with low crystallinity. Calcium aluminate (AFt) manifests as needles and rods, while hydrated calcium silicate (C-S-H) takes on reticulated, colloidal, or flocculated forms. The colloidal structure of C-S-H significantly influences the performance of silicate cements in engineering applications, while the acicular form of AFt contributes to early performance, strength development, and the durability of cementitious materials [42,43]. Calcium aluminate is generally regarded as favorable. Figure 9c,f,i illustrate the abundant hydration products of silicate cement within the soil cement, highlighting soil particles on the fiber surfaces encapsulated by C-S-H. It also showcases AFt connections among particles, leading to a denser soil matrix and enhanced bonding strength between the fibers and the soil matrix.

### 3.4. MIP Pore Structure Analysis

**Figure 11:**
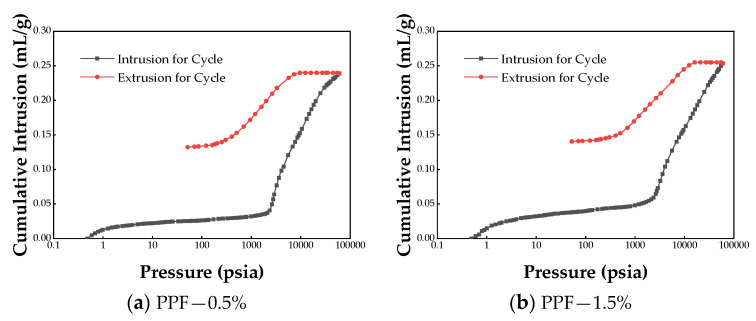

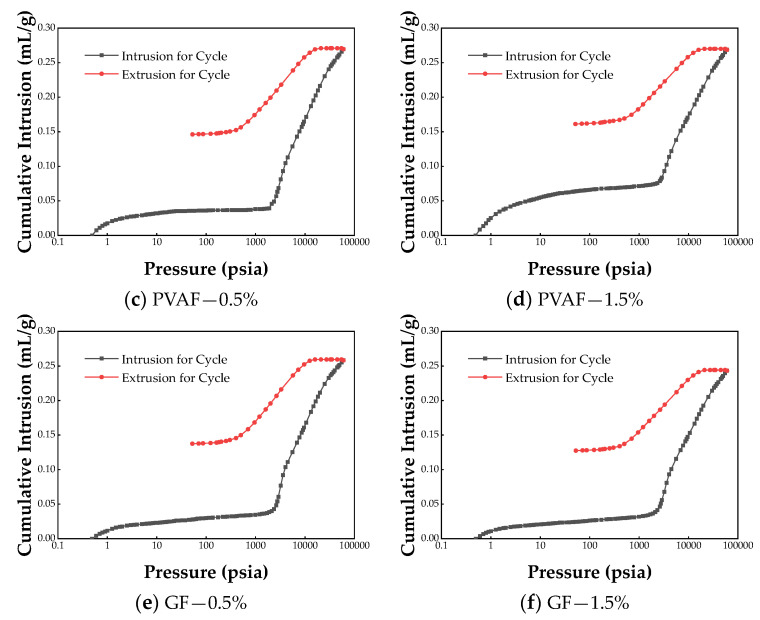
Cumulative intrusion vs. pressure.

**Figure 12:**
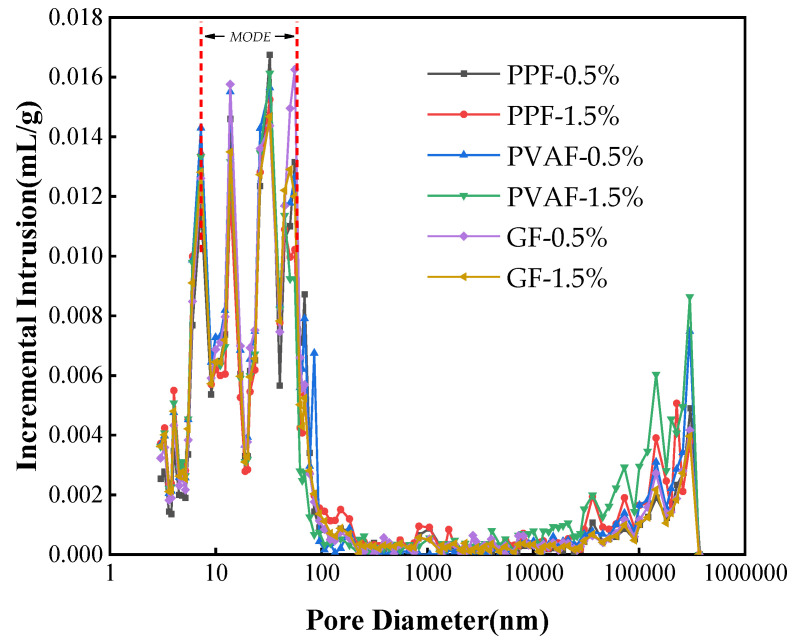
Incremental intrusion vs. pore size.

Mercury intrusion porosimetry (MIP) tests were conducted on fiber-reinforced soil cement specimens with a 0.5% and 1.5% fiber content for each of the three fibers. The inlet and outlet mercury curves derived from the test are depicted in Figure 11. The figure illustrates that the differences between the inlet and outlet mercury curves of the three fiber-reinforced soil cement specimens are significant, indicating a predominance of open pores in these materials and enhanced pore connectivity. With increasing mercury feed pressure, the mercury feed curves of all three types of fiber-reinforced soil cement exhibit a pronounced surge. A significant increase in mercury volume occurs when the pore size reaches a certain threshold, termed the critical pore size, indicating both the connectivity of the pores and the zigzag nature of the infiltration paths [44]. Figure 12 displays the pore size distribution curves for the three fiber-reinforced soil cement specimens. The apex of the pore size distribution curve represents the most prevalent pore size—that is, the pore size most likely to occur. The pore sizes in the samples fall into categories of micropores (<10 nm), small pores (10–100 nm), medium pores (100–1000 nm), and large pores (>1000 nm) [45]. Figure 12 reveals that the most prevalent pore diameters in the three fiber-reinforced soil cement specimens range from 10 nm to 100 nm, indicating a dominance of small pores in these materials. The inlet and outlet curves do not change significantly with the increase in fiber dosage. Similarly, the pore size distribution curves of the three fiber-reinforced soil cement types with varying dosages nearly coincide. In summary, the change in fiber type and dosage has minimal effect on the pore size of soil cement specimens.

MIP measurement is valuable for estimating pore structure parameters, including total porosity and threshold diameter. Cementitious capillary pores consist of elongated percolating chains, with constrictions known as choke points. These constrictions are identified by the diameter known as the threshold diameter. However, MIP has been demonstrated to be unsuitable for accurately measuring the realistic pore size distributions in cementitious materials [46]. Pore size results obtained from MIP often exhibit a bias toward smaller pore sizes due to its measurement methodology, which relies on the diameter of accessible throat pores through which mercury penetrates the microstructure. MIP does not accurately interpret the true pore size distribution; instead, it reflects the size distribution of accessible pores. The accessibility issue, commonly referred to as the ink-bottle effect, significantly hampers MIP’s ability to accurately determine the pore size distribution of cementitious materials. In standard MIP tests, the pressurization procedure compels mercury to penetrate a pore system through throat pores to access interior ink-bottle pores. During the subsequent depressurization procedure, mercury within the throat pores can freely exit, whereas mercury within the interior ink-bottle pores becomes irreversibly trapped [47].

### 3.5. Analysis of Variance (ANOVA)

Two-way ANOVA analyzes the impact of two discrete variables on a continuous variable. In this study, two-way ANOVA was used to determine the effects of fiber type, fiber blend, and their interaction on the unconfined compressive strength and split tensile strength of fiber-reinforced soil cement. Here, fiber type is designated as factor A, and fiber content as factor B. The outcomes of the two-factor analysis are presented in Table 6 and Table 7.

In two-way ANOVA, a factor’s effect is deemed significant if the p-value is less than 5%, indicating 95% confidence [48]. The results from the two-way ANOVA, as detailed in the tables, reveal that the p-values for fiber type, fiber blend, and their interaction significantly fall below the 5% threshold. This indicates that their effects on the unconfined compressive strength and splitting tensile strength of fiber-reinforced soil cement are statistically significant, transcending mere test error.

## 4. Conclusions and Perspectives

In this study, fiber-reinforced soil cement underwent testing for unconfined compressive strength and splitting tensile strength using varying dosages of three types of fibers, PPF, PVAF, and GF, within soil specimens, accompanied by microscopic analyses. The following conclusions were drawn from the study’s findings:

1. The unconfined compressive strengths of the three types of fiber-reinforced soil cement demonstrated a trend of initial increase, followed by a decrease, peaking at a fiber dosage of 0.5%, with respective values of 26.72 MPa, 27.49 MPa, and 27.67 MPa.
2. Similar to the unconfined compressive strength results, the splitting tensile strengths of the three types of fiber-reinforced soil cement exhibited an increasing and then decreasing pattern, peaking at a fiber content of 1.5%, with respective values of 2.29 MPa, 2.34 MPa, and 2.27 MPa.
3. The appropriate incorporation of fibers enhances the total and absorbed elastic strain energy in soil cement, diminishes dissipated energy, and minimizes energy loss.
4. In the unconfined compressive strength test, fibers tend to agglomerate and become entangled when the fiber doping is too high, which results in a decrease in the strength of the soil cement specimen. In the split tensile strength test, the bridging effect of fibers can lead to an increase in the strength of the soil cement specimen. However, a similar phenomenon to that observed in the unconfined compressive strength test occurs when too much fiber is mixed. Therefore, the fiber content varies when the unconfined compressive strength and splitting tensile strength reach their maximum values.
5. The predominant pore diameters in the three types of fiber-reinforced soil cement range from 10 nm to 100 nm, indicating that these specimens mostly consist of small pores.
6. According to this paper, the PVAF-doped fiber-reinforced soil cement performs better compared to the other two fiber-reinforced soil cement types.

This study focused solely on the impact of four dosages of three fiber types on fiber-reinforced soil cement, acknowledging the greater complexity in real-world applications. Future research into other fiber types, varying fiber lengths, and fiber blends is essential.

## Figures and Tables

**Table 1:** Physical and mechanical indexes of kaolin.

Moisture Content (%)	Specific Gravity	Liquid Limit (%)	Plastic Limit (%)	Volumetric Weight (KN/m^3^)
55~60	2.8	77	36	17.2

**Table 2:** Fiber physical and mechanical properties.

Fiber Type	Densities (g/cm^3^)	Diameter (μm)	Tensile Strength (MPa)	Young’s Modulus (GPa)
polypropylene fibers (PPFs)	0.91	21	≥400	≥3.5
polyvinyl alcohol fibers (PVAFs)	1.29	16	≥1550	≥11.5
glass fibers (GFs)	2.70	17.4	≥2000	≥85

**Table 3:** Physical and mechanical parameters of cement.

Specific Surface Area (m^2^/kg)	Standard Consistency (%)	Initial Setting Time (min)	Final Setting Time (min)	Stability	Flexural Strength (MPa)	Compressive Strength (MPa)
368	28.2	184	265	Eligible	5.66	26.3

**Table 4:** Experimental program.

Groups	Fiber Content (%)	Fiber Type
PO0	0	none
PPF0.5	0.5	polypropylene fibers
PPF1	1	
PPF1.5	1.5	
PPF2	2	
PVAF0.5	0.5	polyvinyl alcohol fibers
PVAF1	1	
PVAF1..5	1.5	
PVAF2	2	
GF0..5	0.5	glass fibers
GF1	1	
GF1.5	1.5	
GF2	2	

**Table 5:** Energy parameters of fiber-reinforced soil cement.

Fiber Type	Fiber Content (%)	W (MJ/m^3^)	$W_{e}$ (MJ/m^3^)	$W_{d}$ (MJ/m^3^)
PPF	0	25.63156	24.06238	1.56918
	0.5	27.93692	26.88218	1.05474
	1	26.38991	25.15142	1.23849
	1.5	22.74548	21.23224	1.51324
	2	22.23725	20.98998	1.24727
PVAF	0	25.63156	24.06238	1.56918
	0.5	32.53887	31.26096	1.27791
	1	28.48376	27.02960	1.45416
	1.5	24.72150	23.19695	1.52455
	2	24.68159	22.23571	2.44588
GF	0	25.63156	24.06238	1.56918
	0.5	28.20924	27.47410	0.73514
	1	25.94245	25.45516	0.48729
	1.5	25.42155	24.28560	1.13595
	2	20.79629	19.52832	1.26797

**Table 6:** Two-way ANOVA analysis of compressive strength without lateral limits.

Source	Sum of Squares	Degrees of Freedom	Mean Square	F
A	39.310378	2	19.655189	23.631642
B	98.340915	3	32.780305	39.412108
AB	58.623433	6	9.770572	11.747263
Error	19.961564	24	0.831732	
Total	216.236290	35		

**Table 7:** Two-way ANOVA for split tensile strength.

Source	Sum of Squares	Degrees of Freedom	Mean Square	F
A	0.635871	2	0.317935	18.241834
B	5.794609	3	1.931536	110.823659
AB	0.534335	6	0.089056	5.109660
Error	0.418294	24	0.017429	
Total	7.383109	35		

## Data Availability

All data, models, or codes that support the findings of this study are available from the corresponding author upon reasonable request.
